# Supplementary material for: Feasibility of Employing mHealth in Delivering Preventive Nutrition Interventions Targeting the First 1000 Days of Life: Experiences from a Community-Based Cluster Randomised Trial in Rural Bangladesh
Source: Nutrients. 2024 Oct 10;16(20):3429. doi: 10.3390/nu16203429 (PMC11510744; doi:10.3390/nu16203429)
Supplement: Supplementary file 1 [file nutrients-16-03429-s001.zip › Table S1 Arm-wise Drop-outs.pdf]

Table S1: Arm-wise Drop-outs across ANV 1 to PNV 21m

Intervention visits	Arm 1		Arm 2		Arm 3		Arm 4		Total		P-value
	N	%	N	%	N	%	N	%	N	%	
**Antenatal visits (ANVs)**											
**ANV 1**											
Drop-outs	6	2.4	2	0.8	5	2.0	2	0.8	15	1.5	0.327
Breakdown											
Miscarriage (+abortion)	5	2.0	2	0.8	5	2.0	2	0.8	14	1.4	0.474
Permanent refusal	1	0.4	0	0.0	0	0.0	0	0.0	1	0.1	
**ANV 2**											
Drop-outs	11	4.5	9	3.6	6	2.4	9	3.6	35	3.6	0.676
Breakdown											
Miscarriage (+abortion)	11	4.5	8	3.2	5	2.0	9	3.6	33	3.4	0.776
Permanent relocation beyond study area	0	0.0	1	0.4	1	0.4	0	0.0	2	0.2	
**ANV 3**											
Drop-outs	6	2.6	5	2.1	2	0.8	6	2.5	19	2.0	0.499
Breakdown											
Miscarriage (+abortion)	3	1.3	0	0.0	1	0.4	2	0.8	6	0.6	0.054
Delivered live [eligible for PNV]	2	0.9	0	0.0	1	0.4	1	0.4	4	0.4	
Still birth	0	0.0	0	0.0	0	0.0	2	0.8	2	0.2	
Permanent relocation beyond study area	0	0.0	1	0.4	0	0.0	0	0.0	1	0.1	
Permanent refusal	1	0.4	4	1.7	0	0.0	1	0.4	6	0.6	
**ANV 4**											
Drop-outs	8	3.5	21	9.0	18	7.6	16	6.9	63	6.8	0.119
Breakdown											
Delivered live [eligible for PNV]	6	2.6	19	8.1	16	6.8	12	5.2	53	5.7	0.149
Still birth	2	0.9	1	0.4	1	0.4	4	1.3	8	0.8	

Child death	0	0.0	1	0.4	0	0.0	0	0.0	1	0.1	
Maternal death	0	0.0	0	0.0	1	0.4	0	0.0	1	0.1	
**Postnatal visits (ANVs)**											
**PNV within 48 hours of birth**											
Drop-outs	9	4.0	12	5.3	12	5.1	10	4.4	43	4.7	0.902
Breakdown											
Stillbirth	8	3.5	6	2.6	6	2.5	6	2.6	26	2.8	0.105
Neonatal death	1	0.4	6	2.6	8	3.4	4	1.7	19	2.1	
Permanent relocation beyond study area	0	0.0	1	0.4	0	0.0	0	0.0	1	0.1	
Permanent refusal	1	0.4	3	1.3	0	0.0	1	0.4	5	0.5	
**PNV within 7-14 days of birth**											
Drop-outs	1	0.5	2	0.9	2	0.9	1	0.5	6	0.7	0.882
Breakdown											
Neonatal death	1	0.5	0	0.0	2	0.9	1	0.5	4	0.5	0.070
Permanent refusal	0	0.0	2	0.9	0	0.0	0	0.0	2	0.2	
**PNV 1 month**											
Drop-outs	`	0.0	0	0.0	3	1.4	1	0.5	4	0.5	0.117
Breakdown											
Neonatal death	0	0.0	0	0.0	3	1.4	0	0.0	3	0.3	0.122
Permanent refusal	0	0.0	0	0.0	0	0.0	1	0.5	1	0.1	
**PNV 2 month**											
Drop-outs	2	0.9	1	0.5	0	0.0	3	1.4	6	0.7	0.343
Breakdown											
Infant death	1	0.5	0	0	0	0	0	0	1	0.1	0.435
Permanent relocation beyond study area	0	0	1	0.5	0	0	1	0.5	2	0.2	
Permanent refusal	1	0.5	0	0	0	0	2	0.9	3	0.3	
**PNV 3 month**											

Drop-outs	0	0.0	0	0.0	0	0.0	1	0.5	1	0.1	0.390
Breakdown											
Permanent refusal	0	0.0	0	0.0	0	0.0	1	0.5	1	0.1	0.083
**PNV 4 month**											
Drop-outs*	1	0.5	3	1.4	1	0.5	0	0.0	5	0.6	0.277
Breakdown											
Infant death	1	0.5	1	0.5	1	0.5	0	0.0	3	0.4	0.546
Permanent relocation beyond study area	0	0.0	1	0.5	0	0.0	0	0.0	1	0.1	
Permanent refusal	0	0.0	1	0.5	0	0.0	0	0.0	1	0.1	
**PNV 5 month**											
Drop-outs	0	0.0	0	0.0	0	0.0	0	0.0	0	0.0	…
**PNV 6 month**											
Drop-outs	0	0.0	1	0.5	0	0.0	1	0.5	2	0.2	0.566
Breakdown											
Infant death	0	0.0	1	0.5	0	0.0	0	0.0	1	0.1	0.512
Permanent relocation beyond study area	0	0.0	0	0.0	0	0.0	1	0.5	1	0.1	
Permanent refusal	0	0.0	0	0.0	0	0.0	0	0.0	0	0.0	
**PNV 9 month**											
Drop-outs	1	0.5	1	0.5	1	0.5	3	1.4	6	0.7	0.999
Breakdown											
Infant death	0	0	0	0	0	0	1	0.5	1	0.1	0.879
Permanent relocation beyond study area	1	0.5	1	0.5	1	0.5	1	0.5	4	0.5	
Permanent refusal	0	0.0	0	0.0	0	0.0	1	0.5	1	0.1	
**PNV 12 month**											
Drop-outs	0	0.0	1	0.5	2	0.9	1	0.5	4	0.5	0.581
Breakdown											
Infant death	0	0.0	1	0.5	1	0.5	0	0.0	2	0.2	0.557

Permanent relocation beyond study area	0	0.0	0	0.0	2	0.5	2	1.0	4	0.4	
Permanent refusal	1	0.5	0	0.0	1	0.5	0	0.0	2	0.2	
**PNV 15 month**											
Drop-outs	2	0.9	0	0.0	0	0.0	0	0.0	2	0.2	0.115
Breakdown											
Permanent relocation beyond study area	2	0.9	0	0.0	1	0.5	0	0.0	3	0.4	0.391
**PNV 18 month**											
Drop-outs	0	0.0	1	0.5	1	0.5	0	0.0	2	0.2	0.573
Breakdown											
Permanent refusal	0	0.0	1	0.5	1	0.5	0	0.0	2	0.2	0.064
**PNV 21 month**											
Drop-outs	0	0.0	1	0.5	2	1.0	1	0.5	4	0.5	0.575
Breakdown											
Child death	0	0.0	1	0.5	1	0.5	0	0.0	2	0.2	0.787
Permanent relocation beyond study area	0	0.0	0	0.0	1	0.5	1	0.5	2	0.2	
