# Supplementary material for: Feasibility of Employing mHealth in Delivering Preventive Nutrition Interventions Targeting the First 1000 Days of Life: Experiences from a Community-Based Cluster Randomised Trial in Rural Bangladesh
Source: Nutrients. 2024 Oct 10;16(20):3429. doi: 10.3390/nu16203429 (PMC11510744; doi:10.3390/nu16203429)
Supplement: Supplementary file 1 [file nutrients-16-03429-s001.zip › Table S2 - Arm-wise coverage.pdf]

Table S2: Arm-wise Coverage of ANV 1 to PNV 21m

Intervention visits	Arm 1		Arm 2		Arm 3		Arm 4		Total		P-value
	N	%	N	%	N	%	N	%	N	%	
**Antenatal visits (ANVs)**											
**ANV 1**											
Enrolled women	250	25.0	250	25.0	250	25.0	250	25.0	1000	100.0	
Eligible for ANV 1	244	97.6	248	99.2	245	98.0	248	99.2	985	98.5	0.327
ANV 1 conducted	244	100.0	248	100.0	245	100.0	247	99.6	984	99.9	
**ANV 2**											
Eligible for ANV 2	233	95.5	239	96.4	239	97.6	239	96.4	950	96.4	
ANV 2 conducted	229	98.3	236	98.7	233	97.5	236	98.7	934	98.3	0.676
**ANV 3**											
Eligible for ANV 3	227	97.4	234	97.9	237	99.2	233	97.5	931	98.0	
ANV 3 conducted	222	97.8	221	94.4	229	96.6	221	94.8	893	95.9	0.499
**ANV 4**											
Eligible for ANV 4	219	96.5	213	91.0	219	92.4	217	93.1	868	93.2	
ANV 4 conducted	214	97.7	202	94.8	213	97.3	203	93.5	832	95.9	0.119
**Postnatal visits (PNVs)**											
**PNV Within 48 hours of birth**											
Eligible for PNV within 48 hours of birth	217	95.6	216	93.1	222	94.1	219	95.2	874	94.5	
PNC Within 48 hours of birth conducted	119	54.8	114	52.8	120	54.1	100	45.7	453	51.8	0.077
**PNV within 7-14 days of birth**											
Eligible for PNV within 7-14 days of birth	216	99.5	214	99.1	220	99.1	218	99.5	868	99.3	
Total PNV within 7-14 days of birth conducted	191	88.4	195	91.1	210	95.5	195	89.4	791	91.1	0.882

**PNV 1 month**											
Eligible for PNV 1 month	216	100.0	214	100.0	217	98.6	217	99.5	864	99.5	
PNC 1 month conducted	202	93.5	207	96.7	211	97.2	206	94.9	826	95.6	0.117
**PNV 2 month**											
Eligible for PNV 2 month	214	99.1	213	99.5	217	100.0	214	98.6	858	99.3	
PNC 2 month conducted	204	95.3	210	98.6	210	96.8	208	97.2	832	97.0	0.343
**PNV 3 month**											
Eligible for PNV 3 month	214	100.0	213	100.0	217	100.0	213	99.5	857	99.9	
PNC 3 month conducted	205	95.8	207	97.2	206	94.9	209	98.1	827	96.5	0.390
**PNV 4 month**											
Eligible for PNV 4 month	213	99.5	210	98.6	216	99.5	213	100.0	852	99.4	
PNC 4 month conducted	210	98.6	206	98.1	213	98.6	208	97.7	837	98.2	0.277
**PNV 5 month**											
Eligible for PNV 5 month	213	100.0	210	100.0	216	100.0	213	100.0	852	100.0	
PNV 5 month conducted	210	98.6	206	98.1	211	97.7	206	96.7	833	97.8	
**PNV 6 month**											
Eligible for PNV 6 month	213	100.0	209	99.5	216	100.0	212	99.5	850	99.8	
PNV 6 month conducted	212	99.5	209	100.0	214	99.1	210	99.1	845	99.4	0.566
**PNV 9 month**											
Eligible for PNV 9 month	212	99.5	208	99.5	215	99.5	209	98.6	844	99.3	
PNV 9 month conducted	210	99.1	207	99.5	213	99.1	208	99.5	838	99.3	0.567
**PNV 12 month**											
Eligible for PNV 12 month	211	99.5	207	99.5	212	98.6	207	99.0	837	99.2	
PNV 12 month conducted	208	98.6	207	100.0	210	99.1	204	98.6	829	99.0	0.678

**PNV 15 month**										
Eligible for PNV 15 month	209	99.1	207	100.0	211	99.5	207	100.0	834	99.6	
PNV 15 month conducted	207	99.0	205	99.0	209	99.1	204	98.6	825	98.9	0.306
**PNV 18 month**											
Eligible for PNV 18 month	209	100.0	206	99.5	210	99.5	207	100.0	832	99.8	
PNV 18 month conducted	207	99.0	206	100.0	208	99.0	200	96.6	821	98.7	0.573
**PNV 21 month**											
Eligible for PNV 21 month	209	100.0	205	99.5	208	99.0	206	99.5	828	99.5	
PNV 21 month conducted	205	98.1	203	99.0	205	98.6	203	98.5	816	98.6	0.575

* ANVs open at→ ANV 1: gestational age/GA ≤149days; ANV 2: GA 150days; ANV 3: GA 210days; ANV 4: GA >239days;
PNV 1 to 21m opened at child’s date of birth/DoB + 30 days for each incremental month (e.g. PNV 1m schedule opens at DoB + 30days, PNV 12m at DoB + 365days).
